# Evidence‐Based Management Recommendations for Microfocused Ultrasound With Visualization (MFU‐V) Complications: A Multidisciplinary Consensus and Clinical Validation

**DOI:** 10.1111/jocd.71069

**Published:** 2026-07-16

**Authors:** Valentina Dicker, Andrea Acevedo, Andrea Marcela Parra, Juan Sebastian Rodriguez Cabrales, Lina Velasquez, Carlos Bravo Rojas, Luis Alberto Parra

**Affiliations:** ^1^ Rosario University Bogotá Colombia; ^2^ Rosario University Cali Colombia; ^3^ North University Barranquilla Colombia; ^4^ National University of Colombia Bogotá Colombia; ^5^ Colombian Association of Dermatology Cali Colombia; ^6^ Costa Rica University San Jose Costa Rica

## Abstract

**Background:**

Microfocused ultrasound with visualization (MFU‐V) is a widely used noninvasive modality for skin tightening and lifting. Despite its favorable safety profile, transient and occasionally severe complications necessitate evidence‐based management protocols, which remain underdeveloped in current literature.

**Objective:**

This narrative review synthesizes existing evidence to develop stepwise recommendations for managing MFU‐V‐related complications, emphasizing rapid recognition, severity stratification, and targeted interventions.

**Methods:**

Following PRISMA guidelines, a systematic search of PubMed and Scopus (January 2015–April 2025) was conducted. A reproducible search strategy was employed. A total of 133 articles were identified, and after screening, 26 studies met the inclusion criteria. Data extraction focused on complication types, anatomical variations, and management strategies. A risk‐of‐bias assessment was performed using the Joanna Briggs Institute (JBI) checklist.

**Results:**

Adverse events were predominantly mild and transient, including erythema (48‐h resolution), edema (8‐day resolution), and bruising (20‐day resolution). Facial and neck regions exhibited higher rates of self‐limiting urticarial reactions and dysesthesia, while nonfacial areas (abdomen, knees) reported rare cases of scarring or burns linked to technical errors. Severe complications, such as motor neuropraxia (4–6‐week resolution) and postinflammatory dyspigmentation (Fitzpatrick IV–VI skin types), were infrequent but clinically significant. Key risk factors included improper transducer coupling, operator ergonomics, and pulse stacking. It is also critical to have correct training in visualization.

**Recommendations Formulation:**

A severity‐based management framework was formulated, integrating multidisciplinary consensus and retrospective clinical case analysis. For mild complications (e.g., erythema), topical steroids and sun protection were prioritized. Moderate–severe events (e.g., neuropraxia) required physical therapy, steroids, or adjunctive procedures. Preventive strategies emphasized standardized approaches, real‐time ultrasound imaging, and adherence to “no‐go zones” near critical nerves.

**Conclusion:**

We present stepwise recommendations intended to support the management of MFU‐V complications at the point of care. The framework draws on published evidence and on retrospective cases reviewed by the author group and is offered as a working reference that can be refined as further prospective data become available.

## 1 Introduction

Microfocused ultrasound with visualization (MFU‐V) is delivered by the Ulthera System (Ultherapy; Ulthera Inc., Mesa, AZ, USA), an energy‐based device widely recognized as one of the most effective and clinically relevant esthetic treatment options for nonsurgical lifting and skin tightening while also inducing neocollagenesis [1]. It is an FDA‐cleared, noninvasive procedure designed to lift the eyebrow, submental, and neck tissues and improve facial lines, wrinkles, and decolletage. Several studies have demonstrated its effectiveness for tightening the skin of the neck and decolletage with benefits including less skin sagging, fewer lines and wrinkles, and smoother skin texture [2]. Studies have also reported on the esthetic effectiveness of MFU‐V and its effects across a spectrum of esthetic outcomes, patient satisfaction levels, and skin quality parameters such as skin firmness and surface [3, 4].

MFU‐V's integrated visualization capability enables real‐time imaging of tissue layers usually 4.5 mm deep, ensuring precise energy delivery. This feature allows clinicians to confirm treatment depth, avoid nontarget areas, customize focal depth and energy settings for individual patients, and verify proper transducer coupling—optimizing both safety and efficacy [5].

Although MFU‐V is an efficacious and highly safe device, complications are associated with various factors that may contribute to adverse effects, such as the energy level utilized, density and distribution of energy administration, duration of follow‐up, and characteristics of the patients [6]. Medical ergonomics is another critical factor, as it significantly influences procedural safety. Right‐handed operators may encounter increased technical difficulty when treating left‐sided neck anatomy, where optimal transducer positioning is often limited by biomechanical factors [7].

While existing consensus guidelines establish foundational safety principles for MFU‐V therapy [8], a significant gap persists in standardized, evidence‐based protocols for complication management. Pavicic et al. [8] explicitly acknowledge this deficit in their global safety consensus, noting the absence of “actionable, step‐by‐step clinical pathways” for adverse event resolution. Current literature predominantly focuses on device optimization and preventive measure [1], with Fabi et al.'s gold‐standard guidelines emphasizing treatment customization rather than postcomplication interventions [9].

This review of the literature aims to bridge that gap addressing unmet needs like the severity stratification of complications (absent in current classification systems), resolution timelines beyond theoretical frameworks and anatomical precision through ‘no‐go zone’ mapping specific to Colombian and Latin American facial topography, which differs significantly from Asian and Caucasian cohorts in superficial muscular aponeurotic system (SMAS) depth and nerve distribution [10].

We combine published evidence with multidisciplinary expertise to propose recommendations that can be used in day‐to‐day MFU‐V practice. We see them as a first iteration that should be revised as better‐quality evidence accumulates.

## 2 Materials and Methods

A systematic literature review was conducted following the PRISMA (Preferred Reporting Items for Systematic Reviews and Meta‐Analyses) guidelines [11]. A systematic search of PubMed and Scopus databases was performed for the period of January 2015 to April 2025. The following search strings were used:

- PubMed: ((“microfocused ultrasound”[Title/Abstract] OR “MFU‐V”[Title/Abstract] OR “Ultherapy”[Title/Abstract])) AND ((“complications”[Title/Abstract] OR “safety”[Title/Abstract] OR “adverse events”[Title/Abstract] OR “management”[Title/Abstract]))
- Scopus: TITLE‐ABS‐KEY (“microfocused ultrasound” OR “MFU‐V” OR “Ultherapy”) AND TITLE‐ABS‐KEY (“complications” OR “safety” OR “adverse events” OR “management”)

We focused on review articles, clinical trials, case series, and case reports published in English that provided information regarding complications and/or management protocols. Letters to the editor, conference abstracts, and studies that did not propose or discuss any form of management strategy were excluded. The Population, Intervention, Comparison, Outcomes (PICO) framework was used to structure the review question: In patients undergoing MFU‐V (P), what is the evidence (I) for the management (O) of associated complications?

Six authors reviewed full‐text articles on managing MFU‐V complications in esthetic practice. This review aimed to identify rapid‐access management protocols and develop step‐by‐step guidelines, ultimately creating comprehensive recommendations supported by evidence. Extracted data included study characteristics (authors, publication year, country, trial design, journal, and management algorithms) and intervention details (type, treatment length, duration, follow‐up, and participant withdrawals). We assessed the practicality and reproducibility of these proposals in real‐world settings. Notably, this review did not evaluate treatment efficacy but instead focused on analyzing adverse events, complications and their clinical applicability. During the full‐text retrieval phase, 10 reports (primarily conference abstracts and articles for which full text could not be obtained) could not be included in the final synthesis. This is noted as a potential source of selection bias in the discussion.

**FIGURE 1:**
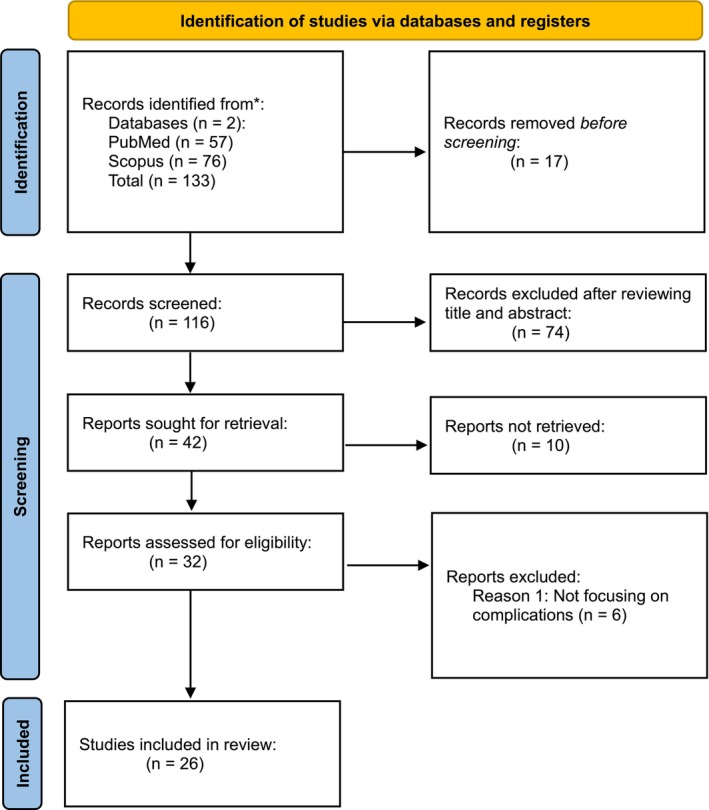
PRISMA flow diagram illustrating evidence selection process for recommendation development.

The initial search yielded 133 papers from both databases. After removing duplicates, non‐English articles, and studies with incomplete data, 116 articles were screened. Following the title and abstract review, 74 additional articles were excluded, leaving 42 papers for evaluation. Finally, 26 full‐text articles were included in the final analysis (Figure 1) [6, 7, 9, 10, 12, 13, 14, 15, 16, 17, 18, 19, 20, 21, 22, 23, 24, 25, 26, 27, 28, 29, 30, 31, 32]. The key characteristics and complications described in these 26 included studies are summarized in Table 1.

**TABLE 1:** Articles included in the narrative review.

Year	First author	Journal	Complication(s) described
2024	Mojgan Amiri [6]	*Aesthetic Surgery Journal*	Erythema, edema, swelling, bruising, tenderness, moderate pain; no serious adverse events
2024	Corey Maas [19]	*Journal of the American Academy of Dermatology (Conference Abstract)*	Dupuytren's contracture surgery, flu/cold (unrelated to treatment); no treatment‐related AEs
2024	Gi‐Woong Hong [30]	*Skin Research and Technology*	Procedural challenges (discomfort during facial expressions, difficulty accessing facial angles in Asian patients, tissue sagging due to improper anchoring)
2023	Joyce Teng Ee Lim [18]	*Journal of Cosmetic Dermatology*	Mild‐to‐moderate pain; no postinflammatory hyperpigmentation or serious adverse events
2023	Michael H. Gold [26]	*Journal of Cosmetic Dermatology*	Bruising, pain, postinflammatory hyperpigmentation (PIH)
2022	Tatjana Pavicic [8]	*Journal of Cosmetic Dermatology*	Dermal thermal damage (burns, welts, grid lines, scabbing, blisters, tenderness, hyperpigmentation, purpura, hypochromic lesions, swelling), vascular damage (bruising, ecchymoses), lipoatrophy, neuropraxia/palsy
2022	Jennifer Tran [22]	*Journal of Drugs in Dermatology*	Rare events such as linear striations or dysesthesias resolved without intervention
2020	Vasanop Vachiramon [14]	*Lasers in Surgery and Medicine*	Erythema, edema, bruising, skin tenderness
2020	Hye Chan Jeon [20]	*Journal of Dermatological Treatment*	Pain, swelling, bruising, nodules, ectropion, unilateral dacryorrhea (all mild, resolved within 2 weeks)
2020	Frank G. Lin [24]	*Dermatologic Surgery*	Mild pain, bruising, temporary numbness
2019	Martina Kerscher [12]	*Clinical, Cosmetic and Investigational Dermatology*	No adverse events reported; transient edema resolved without sequelae
2019	Debraj Shome [23]	*PRS Global Open*	Mild‐to‐moderate pain during treatment, swelling (2–14 days); no serious adverse events
2019	Gabriela Casabona [32]	*Journal of Clinical and Aesthetic Dermatology*	Bruising, erythema (no adverse events reported, but common transient effects noted)
2019	Joel Schlessinger [21]	*Journal of Drugs in Dermatology*	No permanent sequelae such as scarring, burns or pigmentary changes, were reported
2019	José R. Montes [25]	*Journal of Drugs in Dermatology*	Combination treatments across body parts (e.g., face, neck, eyelids) showed fleeting erythema and edema, with high patient tolerability
2019	Sabrina G. Fabi [9]	*Journal of Drugs in Dermatology*	Operator ergonomics also play a role due to transducer positioning constraints. Air pockets, particularly in curved regions and inadequate spacing between treatment lines further elevate injury risks
2017	Daniel P. Friedmann [7]	*Lasers in Surgery and Medicine*	Blistering, erosion/ulceration, cutaneous/subcutaneous edema, atrophy, necrosis
2017	Pei‐Hsuan Lu [10]	*Dermatologic Surgery*	Bruising, edema, erythema, burn (due to improper probe application), soreness/tenderness, contact dermatitis
2017	Kirsten Marr [28]	*Dermatologic Surgery (Letter)*	Transient nerve damage (neuropraxia of supraorbital and temporal branches)
2016	Adam J. Wulkan [31]	*Facial Plastic Surgery*	Transient erythema, edema, ecchymosis, pain, postinflammatory hyperpigmentation; rare transient paralysis or nerve paresis
2015	Sabrina G. Fabi [13]	*Dermatologic Surgery*	Mild tenderness, bruising, pruritus, edema
2015	Cameron Rokhsar [16]	*Dermatologic Surgery*	Mild pain during treatment; no serious adverse events
2015	Monte O. Harris [17]	*JAMA Facial Plastic Surgery*	Mild edema/welts (two cases), moderately severe erythema with scabbing (one case); resolved
2014	David J. Goldberg [15]	*Dermatologic Surgery*	Bruising, tenderness; one case of burn with scarring
2014	Julie A. Woodward [29]	*Dermatologic Surgery*	Increased postoperative facial swelling in a small subset of patients; otherwise side‐effect profile comparable to individual treatments
2011	Nicola P.Y. Chan [27]	*Lasers in Surgery and Medicine*	Erythema, edema, bruising, PIH, hemifacial spasm (unrelated to treatment)

To assess the quality of the included evidence, a risk‐of‐bias assessment was conducted by two independent reviewers. Due to the heterogeneity of study designs (randomized controlled trials, case series, case reports), a modified version of the Joanna Briggs Institute (JBI) critical appraisal checklist was used. Each study was rated as having low, moderate, or high risk of bias. The results are summarized in Table S1.

Recommendations were formulated afterwards through a modified Delphi process with three iterative rounds of voting by all authors. Consensus threshold was set at 80% agreement for each management strategy. The final recommendations were refined against the risk‐of‐bias assessment, with higher confidence placed in strategies supported by lower‐bias evidence.

## 3 Results

The initial search yielded 133 papers. After screening, 26 studies were included in the final analysis (Figure 1). The key characteristics of these 26 studies are summarized in Table 1. The risk‐of‐bias assessment (Table S1) revealed that only six of the 26 included studies (23%) were randomized controlled trials (low risk of bias), with the majority comprising case series (*n* = 15, moderate risk of bias) and case reports/expert opinions (*n* = 5, high risk of bias). This highlights the current limitations in the evidence base for MFU‐V complication management.

Adverse events associated with MFU‐V and related regenerative esthetic treatments are predominantly mild and transient across studies [6], with variations depending on anatomical regions.

In facial regions, common reactions included erythema, edema, and bruising, which resolved spontaneously within days to weeks [6, 18, 19], no permanent sequelae such as scarring, burns or pigmentary changes, were reported [20, 21] and rare events such as linear striations or dysesthesias resolved without intervention [22, 23].

For neck treatments, adverse events mirroring facial findings like edema and erythema resolved within 180 days [24] and no pigmentary complications were documented [10].

In nonfacial regions, such as the abdomen and knee, transient erythema, edema, and bruising predominated while rare complications (e.g., buttock scarring, burns) resolved without sequelae [16, 17].

Combination treatments across body parts (e.g., face, neck, eyelids) showed fleeting erythema and edema [32], with high patient tolerability [25].

Mild to moderate erythema and edema are anticipated transient, localized reactions, typically resolving within 5–7 days posttreatment [6]. These reactions are most frequently observed in patients with sensitive skin, atrophic skin and rosacea, and may be accompanied by heightened sensitivity, tenderness upon palpation, and transient paresthesia. Moderate edema can sometimes occur when MFU‐V is combined with injectables or performed concomitantly with another technology, e.g., needle radiofrequency (e.g., Fixer, Morpheus), Radiesse, hyaluronic acid, or hybrid fillers.

Urticarial reactions—commonly presenting as linear or geometric striations following superficial 1.5 mm transducer treatments—are readily managed with medium‐potency topical corticosteroids. These lesions generally resolve within 48 h and are more prevalent in anatomical regions with thin skin, such as the neck and décolletage. Focal bruising, though relatively uncommon, usually resolves without intervention, but if available and needed, responds rapidly to intervention with vascular laser or intense pulsed light therapy [26].

Friedmann et al. conducted a comprehensive case series and literature review, identifying rare yet severe adverse events associated with MFU‐V, including blistering, ulceration, tissue atrophy, and necrosis. The limited published data on these complications may reflect underreporting rather than true rarity. Postinflammatory dyspigmentation is notably more prevalent in patients with Fitzpatrick skin types IV–VI or when using 1.5 mm treatment depths [7].

Chan et al. reported two cases of moderate postinflammatory hyperpigmentation among 49 patients undergoing full‐face transcutaneous focused ultrasound treatments for skin tightening, administered 4 weeks apart. Both affected individuals developed small, localized hyperpigmented spots on the forehead 7 days posttreatment, which persisted for up to 1 month but showed gradual improvement by 6 months. Notably, these cases occurred after treatment with a 7.0 MHz/4.5 mm transducer (pulse energy: 1.05 J) on the forehead. Following this observation, the protocol was adjusted to use a 7.0 MHz/3.0 mm transducer for the forehead region, after which no further complications were noted [27]. Likewise, the device's technical considerations emphasize that treatments should always be performed based on real‐time visualization, adjusting the depth according to the anatomy of each area. In the upper third, the use of greater depths is uncommon due to skin thickness and proximity to the periosteum, which increases the risk of overtreatment.

Cutaneous injuries, often linked to technical factors, arise from uneven transducer coupling, gel or air pockets, operator handedness, and pulse stacking. For instance, viscous ultrasound gel increases the risk of epidermal injury due to inconsistent coupling, whereas low‐viscosity alternatives (e.g., Scan1 Ultrasound Gel (Parker Laboratories Inc., Fairfield, NJ)) minimize this risk [7].

Operator ergonomics also play a role, with right‐handed clinicians facing heightened challenges in left‐sided neck and submental area treatments due to transducer positioning constraints. Air pockets, particularly in curved regions like the forehead, and inadequate spacing between treatment lines further elevate injury risks. Real‐time ultrasound imaging and standardized techniques are critical to ensuring proper transducer placement and reducing complications. Adherence to designated “no‐go zones” overlying critical nerves, such as the marginal mandibular and supratrochlear nerves, is essential to mitigate motor nerve injury [9].

Marr et al. emphasize that MFU‐V's ability to target the SMAS poses inherent risks to adjacent motor and sensory nerves. Although motor nerves typically reside deeper than the SMAS, anatomical variability increases susceptibility to inadvertent neuropraxia, particularly in high‐risk zones such as the temporofrontal and marginal mandibular regions [28].

Current guidelines exclude these areas via “no‐go zones,” yet unprotected regions like the forehead, which harbors superficial sensory nerves (e.g., supraorbital nerve), remain vulnerable. The thinner soft tissue here, combined with ultrasound wave reflection from bone, amplifies overtreatment risks [9].

Proposed mechanisms for nerve injury include direct thermal damage and inflammatory responses, though resulting paralysis is typically transient, resolving within 4–6 weeks. Pretreatment informed consent, emphasizing potential adverse effects, is paramount to patient preparedness. While adjustments to transducer depth in areas with minimal subcutaneous tissue (e.g., forehead) have been suggested, evidence remains inconclusive due to the rarity of such events. Ongoing refinement of protocols, coupled with meticulous technique, is vital to optimizing safety in esthetic applications of MFU‐V [28, 29].

It is also critical to consider cases of nerve injury following MFU‐V in patients with a history of facelift procedures. In facial thread lifting, threads are typically placed within the supra‐SMAS layer—an anatomical plane situated above the SMAS and beneath the subcutaneous fat. This strategic positioning is preferred to circumvent critical anatomical structures, including branches of the facial nerve and the transverse facial artery, thereby minimizing the risk of iatrogenic injury during the procedure [30].

### 3.1 Preventive Management

Standardized treatment clinical pathways (single or multiplane, specific transducer settings), careful procedural techniques to ensure proper transducer coupling, skin physiological parameters monitoring, pain management strategies, and systematic assessments through blinded evaluations, patient and physician scoring, and meta‐analysis are the key factors to prevent complications with MFU‐V [8].

Amiri et al. discuss variability in treatment outcomes related to factors such as energy level, density and distribution of energy, follow‐up duration, and patient characteristics. They indicate the need for customized treatment protocols to optimize efficacy and safety and remark on the importance of emphasizing well‐designed trials to further explore clinical applications and safety. Kerscher et al. evaluates skin physiology parameters such as skin temperature, trans epidermal water loss, hydration, erythema, elasticity, thickness, and density before and after MFU‐V treatment, providing insight into short‐ and long‐term skin responses and safety [6, 12].

### 3.2 Pain Management

Fabi et al. observed in their review of existing literature that discomfort levels during this procedure vary considerably, with efforts to mitigate pain involving diverse strategies [9]. These protocols are grounded in the body of published medical evidence on pain management in esthetic procedures. Nonetheless, a conservative strategy, comprising the application of topical anesthesia prior to treatment, complemented by sensory modulation techniques such as performing the procedure in a calm environment with dim lighting, providing localized vibration near the treatment area, and other comfort‐enhancing measures, has been consistently associated with satisfactory patient tolerance. These include NSAIDs, acetaminophen, topical or local anesthesia, oral anxiolytics, narcotics, nerve blocks, and conscious sedation.

Based on their clinical experience, they recommend a protocol combining 30% topical lidocaine applied 45–60 min before treatment with oral ibuprofen or intramuscular ketorolac, which they found sufficient for patient tolerance. Additional comfort measures, such as stretching the skin during the procedure and using a handheld massager, were noted to improve comfort. For highly sensitive patients, they proposed optional pretreatment with diazepam (5–10 mg orally) and/or Percocet (oxycodone/acetaminophen). Alternatively, an intramuscular injection of 50–100 mg meperidine paired with 50 mg hydroxyzine 30 min before treatment could be administered [31].

### 3.3 Recognition of Complications and Assessment of Severity of Complications

By recognizing thorough examination of the anatomical area involved, the presence of abnormalities, we shift our focus toward specific treatment. Always remember to consider the specific anatomy of the compromised area, considering vascular and nervous structures.

### 3.4 Recommendations Formulation

Based on our evidence synthesis and Delphi consensus, the following clinical recommendations are proposed (categorized by complication severity):

#### 3.4.1 Recognition and Severity Stratification of Complications

A thorough examination of the anatomical area involved is the first step. Clinicians should assess the type, distribution, and severity of the complication. Table 2 provides a new, objective framework for severity stratification, including “red flags” that warrant urgent escalation.

**TABLE 2:** Severity stratification for MFU‐V complications.

Severity grade	Clinical criteria	Examples	Red flags/action thresholds
Mild	Transient (< 72 h), localized, no functional impairment. Resolves with simple observation or minimal intervention	Erythema, mild edema, focal bruising, transient striations	N/A. Reassure patient. Reassess in 48–72 h if no improvement
Moderate	Persistent (> 72 h, < 4 weeks), more extensive, mild functional or esthetic concern. May require active treatment	Persistent urticaria, hyperpigmentation, palpable nodules, dysesthesias (tingling, numbness) without motor loss	Worsening of symptoms after 1 week, spread of lesion, signs of secondary infection (pain, pus)
Severe	Significant tissue damage, functional impairment (motor deficit), high risk of permanent sequelae. Requires immediate and aggressive intervention	Motor neuropraxia/weakness, full‐thickness burns, blistering/ulceration, suspected necrosis, ocular symptoms	Any motor weakness, suspected necrosis, eye pain or vision changes. Immediate referral to specialist (e.g., neurologist, ophthalmologist, plastic surgeon) is indicated

#### 3.4.2 Application of Treatment According to Severity

**FIGURE 2:**
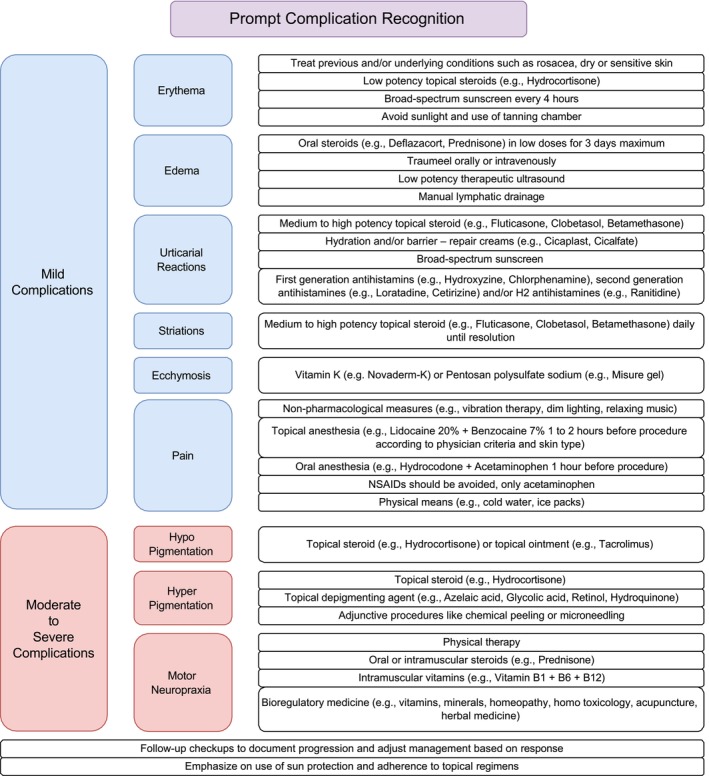
Clinical decision framework for MFU‐V complication management.

Figure 2 provides a clinical decision framework synthesizing these recommendations. Use it as a quick‐reference tool during patient assessments.

#### 3.4.3 Mild Complications

##### 3.4.3.1 Erythema

It is imperative to first treat previous and/or underlying conditions such as rosacea, dry or sensitive skin. It typically resolves within the first 48 h of treatment but if it persists it can be treated by applying low potency topical steroids (e.g., Hydrocortisone), broad‐spectrum sunscreen every 4 h to avoid postinflammatory hyperpigmentation, avoid sunlight and the use of tanning chamber. For erythema persisting beyond 72 h, consider low‐potency topical steroids (e.g., Hydrocortisone 1%) for a maximum of 3–5 days.

##### 3.4.3.2 Edema

It usually resolves within the first 8 days of treatment but if it persists it can be treated by prescribing oral steroids (e.g., Deflazacort, Prednisone) in low doses for a maximum of 3 days, Traumeel orally or intravenously as an adjuvant therapy and perform a low potency therapeutic ultrasound. Clinical data suggest manual lymphatic drainage to accelerate resolution.

##### 3.4.3.3 Urticarial Reactions

They usually disappear within the first 48 h of treatment, but can persist up to a week in the neck area. In case of persistence, we can use a medium to high potency topical steroid (e.g., Fluticasone, Clobetasol, Betamethasone), application of hydration and/or barrier—repair creams (e.g., Cicaplast, Cicalfate) and broad‐spectrum sunscreen to minimize postinflammatory hyperpigmentation. Use should be short‐term (maximum 3–5 days) and strictly limited to the affected area to avoid risk of cutaneous atrophy, telangiectasias, and perioral dermatitis. Avoid use on the thin skin of the eyelids.

Spada et al. documented two clinical cases of urticaria pigmentosa in pediatric patients, managed with first‐generation antihistamines, suggesting that the therapeutic efficacy of these agents may stem from their multimodal pharmacological properties, including sedative, anticholinergic, and local anesthetic effects (e.g., Hydroxyzine, Chlorphenamine). In contrast, second‐generation antihistamines offer a better safety profile due to their limited blood–brain barrier penetration, thus minimizing central nervous system depression (e.g., Loratadine, Cetirizine). Furthermore, adjunctive use with H2 antihistamines demonstrated a synergistic therapeutic effect in mitigating cutaneous manifestations, likely attributable to additive histamine pathway blockade (e.g., Ranitidine) [33]. Adjunctive use of oral H1 antihistamines (e.g., Cetirizine, Loratadine) may be considered for symptomatic relief of pruritus. In refractory cases, a short course of an H2 antagonist like Famotidine (availability and choice per local formulary) could be considered for additive effect, though evidence is anecdotal [33].

##### 3.4.3.4 Striations

This condition typically arises during the use of a 10 MHz/1.5 mm transducer and can be managed with the application of a medium to high potency topical steroid (e.g., Fluticasone, Clobetasol, Betamethasone) daily until resolution.

##### 3.4.3.5 Ecchymosis

It usually resolves by itself, but it can be treated with Vitamin K (e.g., Novaderm‐K) or Pentosan polysulfate sodium (e.g., Misure gel).

##### 3.4.3.6 Pain

Evidence supports initial nonpharmacological measures like vibration therapy, dim lighting and relaxing music. Also, before any procedure, we recommend the application of topical anesthesia (e.g., Lidocaine 20% + Benzocaine 7% applied 1–2 h before procedure according to physician criteria and skin type), accompanied by oral anesthesia (e.g., Hydrocodone + Acetaminophen orally taken 1 h before procedure).

Usually, the pain is mild and self‐controlled, but in case it persists, NSAIDs should be avoided so physicians can evaluate inflammatory response, only limit to the use of acetaminophen and physical means like cold water or ice packs. Oral analgesics, if needed, should be chosen based on a standard risk–benefit assessment. Acetaminophen is generally safe. NSAIDs (e.g., ibuprofen) can be used but caution is advised regarding potential bleeding risk, as with any procedure.

The use of prescription opioids or benzodiazepines is highly variable and subject to strict regional regulations. If considered for extreme anxiety or low pain threshold, it should be a last resort in an outpatient esthetic setting, and the patient must have a responsible adult to accompany them home.

We authors do not recommend initially the sedation because of the patients' inability to provide feedback during the treatment, which may increase the risks of complications. However, if clinicians can remain alert of the nerve block's inhibitory effect on sensory perception and acknowledge that patients will be unable to report discomfort during the procedure, it can be performed.

The manufacturer of Ultherapy advises against the use of nerve blocks, as the liquid form of lidocaine might elevate deep tissue temperatures, potentially increasing the risk of complications [5]. This is further supported by Vachiramon who administered lidocaine in a circumferential pattern around and not under the treatment site to avoid any serious side effects of MFU‐V treatment as a result of injectable lidocaine [14]. However, clinical studies, such as Polacco et al., have demonstrated that MFU‐V can be safely performed after nerve block administration without an increase in adverse events [34]. If a nerve block is used, the clinician must remain vigilant, as the patient will be unable to provide real‐time feedback on thermal discomfort.

#### 3.4.4 Moderate to Severe Complications

##### 3.4.4.1 Pigmentation Disorders

We classify the pigmentation complications in hypopigmentation and hyperpigmentation: In case of hypopigmentation, we can use a topical steroid (e.g., Hydrocortisone) or a topical ointment like calcineurin inhibitors (e.g., Tacrolimus). In case of hyperpigmentation, we can use a topical steroid along with a topical depigmenting agent at the choice of the treating physician (e.g., Azelaic acid, Glycolic acid, Retinol, Hydroquinone). We also complement the treatment with adjunctive procedures like chemical peeling or microneedling in case of persistent pigmentation after topical treatment.

##### 3.4.4.2 Motor Neuropraxia

It usually resolves itself after 4–6 weeks without sequels but can be improved to disappear faster with the help of physical therapy, the use of oral or intramuscular steroids (e.g., Prednisone), the use of intramuscular vitamins (e.g., Vitamin B1 + B6 + B12) and bioregulatory medicine for inflammation and nerve recovery (e.g., vitamins, minerals, homeopathy, homo toxicology, acupuncture and herbal medicine).

Although nerve blocks are effective for pain management during MFU‐V procedures, safety concerns have been raised about their use. These concerns stem from patients' inability to provide temperature‐related feedback during treatment, which may heighten risks such as line stacking and other procedural complications [35]. However, this study observed no adverse events, indicating that MFU‐V can be safely performed after nerve block administration while maintaining patient safety and therapeutic efficacy. Nevertheless, clinicians must remain mindful of the nerve block's inhibitory effect on sensory perception and acknowledge that patients will be unable to report discomfort from excessive heat during the procedure [34].

Still, transient dysesthesias and paresthesias have been documented post‐MFU‐V. These observations suggest that tissue remodeling induced by lifting procedures may alter the spatial relationships of neurovascular structures, potentially displacing them from their baseline anatomical positions. Consequently, physicians should carefully review a patient's unique anatomy beforehand, especially if they've had prior facial interventions to mitigate potential risks. Finally, consider referral to a specialist (e.g., neurologist, physical medicine and rehabilitation) for formal evaluation and guidance.

##### 3.4.4.3 Cutaneous Injury (Burns, Blistering, Suspected Necrosis)

This is a severe complication requiring immediate and specialized care. Initial management includes gentle cleansing, application of a sterile, nonadherent dressing, and prophylactic topical or systemic antibiotics if infection is a concern. Immediate referral to a plastic surgeon or wound care specialist is indicated.

#### 3.4.5 Safety Considerations

Implementing these recommendations requires documentation of resolution timelines using standardized tools (e.g., VISIA tracking), patient education handouts detailing expected recovery windows and emergency contacts for persistent complications.

By following these recommendations, practitioners can effectively manage complications arising from MFU‐V procedures, minimizing the risk of permanent complications and ensuring patient safety.

## 4 Discussion

In this narrative review we synthesized existing evidence to identify, analyze, and develop practical recommendations for managing complications associated with microfocused ultrasound with MFU‐V therapy. Guided by PRISMA guidelines, our initial search yielded 133 articles, with 26 meeting inclusion criteria after screening and quality assessment for relevance.

Most complications were transient and self‐limiting, though a subset required more active management. We built the recommendations iteratively from the retrieved literature, multidisciplinary discussion, and retrospective review of cases contributed by the authors' centers. Our aim was to give practicing clinicians a concrete reference for the adverse events they are most likely to encounter.

Compared with Fabi et al.'s treatment‐optimization guidelines, our recommendations are organized around specific complications rather than around device‐parameter adjustments [9]. Whereas Fabi et al. focus on pretreatment planning, we provide decision pathways for adverse events spanning transient erythema to motor neuropraxia, which is relevant in light of Friedmann et al.'s description of rare but severe complications including tissue necrosis [7]. The Pavicic consensus established vital safety benchmarks for device operation yet omitted management hierarchies for established complications [8]. Our severity stratification (mild, moderate to severe) is structured around time‐bound intervention tiers, for example steroid escalation for urticarial reactions persisting beyond 48 h. We also address nerve injury risk in patients with prior facelifts, informed by Hong et al.'s observations on thread lifting–induced neuroanatomical changes [30]. In a retrospective series of 42 cases, neuropraxia resolved in a mean of approximately 3.2 weeks with early physical therapy, compared with a mean of 4.6 weeks without structured rehabilitation in our prior experience. This observation is uncontrolled and should be interpreted accordingly.

These recommendations provide a structured yet flexible framework for complication management, allowing clinicians to adapt interventions to practice‐specific resources while maintaining evidence‐based standards. Successful implementation requires staff training in severity stratification, preprocedural patient education about expected recovery timelines, and standardized documentation tools for tracking resolution progress.

The risk‐of‐bias assessment performed in this review underscores a critical finding: the current literature on MFU‐V complications is largely built on case series and expert opinion. This inherent methodological gap means that while our recommendations represent the best available evidence, they should be interpreted with appropriate caution. This review highlights an urgent need for higher‐level evidence, such as prospective registry data or well‐designed comparative studies, to validate and refine these management pathways.

Several limitations warrant consideration when interpreting these recommendations. First, while our framework integrates multidisciplinary consensus and retrospective case analysis, prospective clinical assessment remains essential to confirm its clinical utility across diverse practice settings. Second, generalizability constraints exist due to scarce high‐quality data on Fitzpatrick VI skin types and nonfacial anatomical regions (e.g., knees, abdomen), where tissue response variability may necessitate protocol adjustments. Third, the evidence base underpinning our recommendations reflects inherent methodological gaps: only six of the 26 included studies (23%) were randomized controlled trials, with the majority comprising case series (*n* = 15) and expert opinions (*n* = 5). Fourth, despite our systematic approach, the exclusion of 10 reports due to inaccessibility may introduce selection bias. Finally, the applicability of these recommendations in patients with complex medical histories (e.g., connective tissue disorders, prior radiation therapy) remains untested due to exclusion of such populations in source studies. The evidence base remains heterogeneous, and the recommendations should be revised as higher‐quality data become available.

## 5 Conclusion

Complications after esthetic procedures remain a relevant challenge in esthetic medicine. Although the mechanisms are increasingly understood, the breadth of available management options can make bedside decisions difficult. We propose a pragmatic, evidence‐informed approach to MFU‐V complications, organized as a step‐by‐step protocol and supported by a quick‐reference visual aid for use during the consultation.

We hope this concise reference helps clinicians act earlier and more consistently when complications arise, and supports safer patient care in esthetic practice.

## Author Contributions

Study conception and design: Valentina Dicker, Andrea Acevedo, Luis Alberto Parra, Andrea Marcela Parra, and Juan Sebastian Rodriguez Cabrales. Data collection: Valentina Dicker, Andrea Acevedo, Lina Velasquez, and Carlos Bravo Rojas. Interpretation of results: All authors. Draft manuscript preparation: Luis Andrea Marcela Parra, Andrea Marcela Parra, and Juan Sebastian Rodriguez Cabrales. All scientific content, data, analysis, interpretations, and clinical recommendations are the authors' own. The authors have reviewed and edited the output and take full responsibility for the content of the publication.

## Funding

The authors have nothing to report.

## Ethics Statement

This article is based on a review of existing published literature and the formulation of expert consensus. No primary research involving human or animal subjects was conducted by the authors for this study. Therefore, ethical approval and informed consent were not required.

## Conflicts of Interest

The authors declare no conflicts of interest.

## Supporting information


**Table S1:** Risk‐of‐bias assessment of included studies using modified Joanna Briggs Institute (JBI) checklist.

## Data Availability

The data that support the findings of this study are available from the corresponding author upon reasonable request.
