# Supplementary material for: Evidence‐Based Management Recommendations for Microfocused Ultrasound With Visualization (MFU‐V) Complications: A Multidisciplinary Consensus and Clinical Validation
Source: J Cosmet Dermatol. 2026 Jul 16;25(7):e71069. doi: 10.1111/jocd.71069 (PMC13376685; doi:10.1111/jocd.71069)
Supplement: Supplementary file 1 — Table S1: Risk‐of‐bias assessment of included studies using modified Joanna Briggs Institute (JBI) checklist. [file JOCD-25-e71069-s001.docx]

| **Citation** | **Design** | **Q1** | **Q2** | **Q3** | **Q4** | **Q5** | **Q6** | **Q7** | **Q8** | **Q9** | **Q10** | **Appraisal note** |
| --- | --- | --- | --- | --- | --- | --- | --- | --- | --- | --- | --- | --- |
| Amiri | Systematic review and meta analysis | N/A | N/A | N/A | N/A | N/A | N/A | N/A | N/A | N/A | N/A | Secondary evidence review |
| Maas | Prospective interventional study | Yes | Yes | Yes | Yes | No | No | Yes | Yes | N/A | N/A | Single arm acne scar study |
| Hong | Narrative review | N/A | N/A | N/A | N/A | N/A | N/A | N/A | N/A | N/A | N/A | Off topic narrative review on thread lifting |
| Lim | Uncontrolled pilot study | Yes | Yes | Yes | Yes | No | No | Yes | Yes | N/A | N/A | Uncontrolled pilot study |
| Gold | Off topic cellulite bruising article | N/A | N/A | N/A | N/A | N/A | N/A | N/A | N/A | N/A | N/A | Listed in manuscript references but not an MFU V clinical study |
| Pavicic | Consensus review | N/A | N/A | N/A | N/A | N/A | N/A | N/A | N/A | N/A | N/A | Consensus and review article |
| Tran | Prospective split face open label study | Yes | Yes | Yes | Yes | No | Yes | Yes | Yes | No | No | Open label split face pain study |
| Vachiramon | Randomized comparative study | Yes | Yes | Yes | Yes | No | Yes | Yes | Yes | Yes | No | Randomized comparison without clear blinding |
| Jeon | Retrospective cohort study | Yes | Yes | Yes | No | Yes | Yes | Yes | Yes | N/A | N/A | Retrospective analysis with multivariable model |
| Lin | Prospective single arm study | Yes | Yes | Yes | Yes | No | No | Yes | Yes | N/A | N/A | Single arm postpartum abdominal study |
| Kerscher | Observational prospective study | Yes | Yes | Yes | Yes | No | No | Yes | Yes | N/A | N/A | Observational physiology study |
| Shome | Prospective case series | Yes | Yes | Yes | Yes | No | No | Yes | Yes | N/A | N/A | Prospective case series |
| Casabona | Prospective adjunctive single arm study | Yes | Yes | Yes | Yes | Yes | No | Yes | Yes | N/A | N/A | Adjunctive striae study after prior treatments |
| Schlessinger | Randomized dose comparison trial | Yes | Yes | Yes | Yes | No | Yes | Yes | Yes | Yes | No | Randomized treatment density study |
| Montes | Retrospective cross sectional survey | Yes | Yes | Yes | No | No | No | Yes | Yes | N/A | N/A | Survey based satisfaction study |
| Fabi | Consensus guideline | N/A | N/A | N/A | N/A | N/A | N/A | N/A | N/A | N/A | N/A | Expert consensus guideline |
| Friedmann | Case series plus literature review | Yes | Yes | Yes | Yes | No | No | Yes | N/A | N/A | N/A | Case series focused on complications |
| Lu | Prospective single arm study | Yes | Yes | Yes | Yes | No | No | Yes | Yes | N/A | N/A | Single arm efficacy study |
| Marr | Case report or case series | No | Yes | Yes | Yes | No | No | Yes | N/A | N/A | N/A | Case report or small case series on adverse event |
| Wulkan | Narrative review | N/A | N/A | N/A | N/A | N/A | N/A | N/A | N/A | N/A | N/A | Narrative review |
| Fabi | Prospective multicenter pilot study | Yes | Yes | Yes | Yes | No | No | Yes | Yes | N/A | N/A | Single arm multicenter pilot |
| Rokhsar | Prospective single arm study | Yes | Yes | Yes | No | No | No | Yes | Yes | N/A | N/A | Single arm body area study |
| Harris | Open label nonrandomized trial | Yes | Yes | Yes | Yes | No | No | Yes | Yes | N/A | N/A | Open label safety trial |
| Goldberg | Prospective single arm study | Yes | Yes | Yes | No | No | No | Yes | Yes | N/A | N/A | Single arm body area study |
| Woodward | Prospective combination study | Yes | Yes | Yes | No | No | No | Yes | Yes | N/A | N/A | Combination treatment study |
| Chan | Prospective safety study | Yes | Yes | Yes | No | No | No | Yes | Yes | N/A | N/A | Early prospective safety study |

**Supplementary Table S1:** Risk-of-Bias Assessment of Included Studies Using Modified Joanna Briggs Institute (JBI) Checklist
